# The American Dental Weekly

**Published:** 1898-04

**Authors:** 


### The American Dental Weekly.

A weekly dental journal was established on September 9, 1897, at Atlanta, Ga., by Dr. B. H. Catching.

The projecting of an enterprise of this sort certainly indicates vim and pluck on the part of the editor and owner, and especially so since other efforts of the same sort have been inaugurated in the past which did not receive a sustaining support, and were forced out of the field.

We have always given Dr. Catching credit for energy and perseverance considerably above the lot of ordinary men, but we must confess that when this effort was proposed and the work put in operation that there was a little bit of misgiving as to success; however, as the journal appears from week to week, this misgiving has been fading away till now it is about all gone, and we entertain strong confidence that this will be one of the permanent, efficient and valued journals of the profession.

There are quite a number of weekly medical journals, and some of them of quite large proportions; some of these have been well sustained for many years.

There is no questisn but that there is room in the profession for a weekly dental journal, and Dr. Catching seems to be just the man for this work. The numbers thus far have been filled with interesting and valuable material, that which will be of real service to the busy practitioner. We most heartily commend this journal to the profession.

